# Optimal Placement of Social Digital Twins in Edge IoT Networks

**DOI:** 10.3390/s20216181

**Published:** 2020-10-30

**Authors:** Olga Chukhno, Nadezhda Chukhno, Giuseppe Araniti, Claudia Campolo, Antonio Iera, Antonella Molinaro

**Affiliations:** 1 DIIES Department, University Mediterranea of Reggio Calabria, 89100 Reggio Calabria, Italy; olga.chukhno@unirc.it (O.C.); nadezda.chukhno@unirc.it (N.C.); araniti@unirc.it (G.A.); claudia.campolo@unirc.it (C.C.); 2 Faculty of Information Technology and Communication Sciences, Tampere University, 33720 Tampere, Finland; 3 Institute of New Imaging Technologies (INIT), Universitat Jaume I, 12071 Castelló de la Plana, Spain; 4 DIMES Department, University of Calabria, 87036 Rende, Italy; antonio.iera@dimes.unical.it; 5 Laboratoire des Signaux et Systémes (L2S), CentraleSupélec, Université Paris-Saclay, 91190 Gif-sur-Yvette, France

**Keywords:** Internet of Things, Social Internet of Things, edge computing, digital twin, optimization problem

## Abstract

In next-generation Internet of Things (IoT) deployments, every object such as a wearable device, a smartphone, a vehicle, and even a sensor or an actuator will be provided with a digital counterpart (twin) with the aim of augmenting the physical object’s capabilities and acting on its behalf when interacting with third parties. Moreover, such objects can be able to interact and autonomously establish social relationships according to the Social Internet of Things (SIoT) paradigm. In such a context, the goal of this work is to provide an optimal solution for the social-aware placement of IoT digital twins (DTs) at the network edge, with the twofold aim of reducing the latency (i) between physical devices and corresponding DTs for efficient data exchange, and (ii) among DTs of friend devices to speed-up the service discovery and chaining procedures across the SIoT network. To this aim, we formulate the problem as a mixed-integer linear programming model taking into account limited computing resources in the edge cloud and social relationships among IoT devices.

## 1. Introduction

A wide variety of Internet of Things (IoT) objects ranging from personal devices (such as, for example, body sensors, high-, medium- and low-end wearable devices, consumer devices, etc.) to intelligent measuring devices, sensors, and even actuators is massively used in smart environments. Such a remarkable rise in connected devices has caused a severe growth of mobile data traffic [1], contributing to the advent of the fifth-generation (5G) system, which is to be rolled out worldwide in the upcoming years. This brings explosive growth opportunities for novel IoT applications leveraging the opportunity of exchanging data with every object with a minimum level of computation and storage capabilities. According to the recent forecast in [2], the number of IoT connections will reach 24 billion by the end of 2025.

The Social Internet of Things (SIoT) paradigm [3,4] has gained ground in recent literature as a valuable means to integrate humans and machines into one global social network and to allow IoT heterogeneous devices to interact, share, and exchange data by taking into account both their physical constraints and their “social” behavior.

The combination of social networking concepts with the IoT offers a number of advantages. First, SIoT enables effective discovery of the services offered by IoT objects, based on typical social network mechanisms. Second, it allows the exchange on a social basis of information associated with/generated by devices, also through new group-based communication schemes like *sociocast* [5,6]. Third, it ensures scalability through social collaboration among nodes. A further remarkable beneficial aspect is the possibility to establish social relationships between objects that use different technologies; this allows device interoperability across different IoT platforms. Finally, SIoT can leverage the degree of interaction between objects to guarantee trusted connections between friend devices [7].

However, IoT devices are typically resource-constrained, hence creating and managing social relationships would further challenge their design. A widespread adoption of IoT, even more when the social dimension is considered, is unlikely without embracing the concept of cloud computing. Indeed, the cloud can complement resource-constrained IoT devices with additional computing, storage, and context-awareness capabilities. In particular, the *digital twin* (DT) [8,9] concept has gained momentum as a concrete means to implement such a vision by bridging the physical world with the digital one. Physical objects are augmented with a digital footprint hosted in the remote cloud and, thus, enabled to perceive the environment better and understand their role in the context in which they are immersed.

Unlike the existing literature [10,11,12], in this work, we align the DT concept with the edge computing paradigm [13], among the most important technological trends that dominate the IoT market in 2020 [14]. Edge computing [15,16,17] aims to localize processing resources closer to end-devices rather than to a centralized cloud computing environment. Since data do not traverse over a network to the cloud to be processed, the network load and the data latency are significantly reduced. This enables faster response times and improved user satisfaction, while also benefiting network operators and cloud providers.

As a further advancement w.r.t. state-of-the-art [18,19], we consider DTs of physical objects enhanced with social capabilities, namely Social DTs (SDTs). Early germs of such an approach can be found in [20,21], where the concept of the Social Virtual Entity (SVE) is preliminarily introduced.

Notwithstanding, hosting applications and SDTs in particular, at the edge, raise several challenges to be addressed. Some of them are generic issues inherited from the literature dealing with the placement of virtualized applications [22,23,24] at the edge; others specifically arise due to the SIoT environment. First, resources at the network edge are limited and unevenly distributed among the available edge servers colocated with access points (APs), base stations (BSs), etc. Second, low-latency connectivity may need to be ensured between the physical and the corresponding virtual counterparts to fulfill the DT objectives. This is especially true when interactive applications are considered. Third, SDTs may also need to be linked among each other in view of a quick navigation of the social network of IoT devices, whenever resources or capabilities of friend devices (e.g., in the event of a road traffic accident or a disaster, for health monitoring, and even for infotainment in public places) need to be discovered and/or chained.

In such a context, the paper aims to provide the following main contributions:

- propose a framework for the Social-aware Closest Edge Placement (SoCEP) of DTs that builds upon the edge computing and SIoT paradigms;
- formulate the SDT placement as a Mixed Integer Nonlinear Programming (MINLP) problem. The proposal accounts for the limited computing resources of edge servers, social relationships among IoT devices, and constraints on the latency in the connectivity between a physical device and the corresponding DT and in the inter-DT connectivity;
- further transform the MINLP problem into a Mixed Integer Linear Programming (MILP) problem by linear relaxation;
- evaluate the performance of the proposal against a baseline solution, which places the DT at the closest edge server and is agnostic w.r.t. social relationships, under different settings in terms of storage constraints on edge servers and latency demands.

The paper is structured as follows. Section 2 gives a brief overview of background material for our work by scanning the literature related to edge computing, digital twins, and SIoT. A general overview of the proposed SoCEP framework is outlined in Section 3. Section 4 focuses on the mathematical formulation of the proposal as an optimization problem. In Section 5, simulation experiments and comparative analysis are presented. Final remarks and conclusions are drawn in Section 6.

## 2. Background and Motivations

### 2.1. Edge Computing for IoT

As most IoT objects are resource-hungry, the gap between required and locally available resources widens and, in this context, cloud computing is proposed as a valuable means to overcome the problem. However, some IoT applications require short response times (e.g., automation control in a smart factory), others produce vast volumes of data to be processed (e.g., augmented reality), whereas some of them may also demand security guarantees (e.g., surveillance in a public place). Cloud computing cannot satisfy these requirements and fails to support this type of IoT applications. By hosting storage and processing resources, as well as applications, closer to the end-users, edge computing can handle the aforementioned issues [25].

In recent years, there has been considerable interest in edge computing for IoT [26,27], also fueled by the activities in the European Telecommunications Standards Institute (ETSI), which refers to such a paradigm as multiaccess edge computing (MEC) [17,28].

A large body of literature addresses computing offloading [29,30] in the view of allowing IoT devices to speed up data processing, thus reducing energy consumption. Among others, in [31], the problem of selecting the appropriate offloading routes for the IoT services is addressed. The optimization of the response time of IoT applications is done in [32,33], where computation offloading methods improve user experience in terms of latency.

Further, edge computing opens up new opportunities in the field of IoT that would not be possible by leveraging traditional cloud-based systems. For instance, Digital Twins, which have already been shifted from concept to reality [19], can be effectively implemented at the edge. The idea of DTs was first introduced in [34] and later formalized in [10], where the main elements of the DT concept are identified, namely, a real space (physical objects), a virtual space (virtual objects), and the link for the data flow between real and virtual domains. Virtual objects provide the semantic description of the related physical objects and of their resources (e.g., memory, storage, processing) and capabilities (e.g., sensing, actuation, computing), which are abstracted into a set of attributes. This abstraction allows performing an effective search of the capabilities/resources needed for creation and composition of IoT services at the application layer [20].

The virtualization layer has become a key component of many reference IoT platforms (e.g., iCore [35], IoT-A [36]) and commercial implementations (e.g., Amazon Web Services IoT) [20], and the interest for it has been further boosted by the emerging concept of digital twins [37]. However, as recently investigated in [19], the communication processes between a physical object and its virtual counterpart and among different DTs are still open issues attracting considerable interest, as well as the proper placement of such virtual entities at the edge to cope with the distributed and limited nature of computing and storage resources of edge servers.

A growing body of literature has investigated the possibilities of edge networks to satisfactorily meet the latency constraints on pairing a physical device and its DT [21,38]. A cost-aware cloudlet placement strategy accounting for the cost of deploying edge servers and the end-to-end latency between physical objects and their avatars is proposed in [24]. In [39], the placement problem is considered as a generalized assignment problem to reduce latency. Further related works focus on virtual machine replica placement problem [40,41], service entity problem [23,42], and joint service placement [43,44] with additional focuses on, e.g., request routing/scheduling [22,45].

### 2.2. SIoT Basics

In the past years, the concept of using elements of social networks in IoT has attracted an unprecedented amount of attention of the research community [46,47,48,49,50]. The synergy of social networking and IoT paradigms can offer several benefits and allow the devices to create relationships in an autonomous way.

The paradigm of “social network of intelligent objects” was proposed in [3]. SIoT aims to simplify the navigability of a network of billions of devices and to enhance their trustworthiness. The key idea of SIoT consists in enabling devices to create their social network and effectively search through social links for the desired services offered by their friend devices [51]. Social relationships can be created, for instance, between objects belonging to the same owner, between fixed devices located in the same place, between objects carried by people who meet frequently, between objects of the same model, vendor, and production batch. SIoT supports many novel enthralling applications and services for the IoT in a more powerful and productive [52] way by facilitating the interaction between physical objects through the digital world. In particular, the exploitation of social network principles in the IoT domain has proven to foster resource visibility, enhance device and service discovery, and enable practical object reputation assessment, service composition, and source crowding [53,54].

Researchers have followed various approaches to design a SIoT architecture and implement a platform for the construction of a SIoT service environment and the virtualization of applications [52,55]. In [56], a technique for the implementation of Virtual Entities (virtual equivalents of the physical objects [20]) is addressed. In [57], a cloud-based social IoT solution, wherein each physical device has a virtual counterpart, is developed. The platform has four major features, i.e., social agents, Platform as a Service model, reusability, and cloud storage. An analogous approach is presented in [58], where virtual objects of physical devices hosted at the edge are enabled to browse the social network of devices.

Indeed, IoT applications can be built in order to exploit data and resources provided by friends. For instance, some applications may need to push (query) data to (from) categories of friends [21]. Moreover, a composite service may be instantiated through the chain of resources (e.g., cached data) provided by the SDTs of friend devices. In these special cases endowed by social flavor, the discovery of resources/capabilities of friend devices over the social network may be facilitated by the presence of social DTs at the edge, which expose them on behalf of the physical devices. Hence, applications that may need to quickly browse the social network by visiting SDTs of friend devices could benefit from finding SDTs of friends either in the same or nearest edge servers. This would result in a short latency and in a reduction of the amount of data traversing the edge infrastructure.

### 2.3. Motivations and Objectives

The surveyed literature shows that prior research efforts (i) identified DTs as a prominent solution for IoT and also for SIoT and (ii) recognized the need to push their deployment at the edge. However, they fall short of addressing the proper placement of DTs, augmented with a social dimension, in network edge deployments with multiple servers.

In order to fill this gap, we propose a framework for the *social-aware placement* of DTs at the network edge. By this meaning that, on the one hand, we address the placement problem by considering the common proximity-driven approach for pairing physical devices and the corresponding DTs [24]; on the other hand, we also target to minimize the latency experienced between edge servers hosting DTs, for which the corresponding physical devices have a social relationship. This is meant to ensure that SIoT objects can quickly communicate and discover services querying their social relationship network on the virtualization layer hosting digital twins.

## 3. System Overview

In this section, our framework for the social-aware placement of digital twins in Edge IoT networks is presented. We define the architecture and introduce the system model for SDTs placement management. The main notations employed in this paper are summarized in Table 1.

### 3.1. Reference Architecture

**Figure 1:**
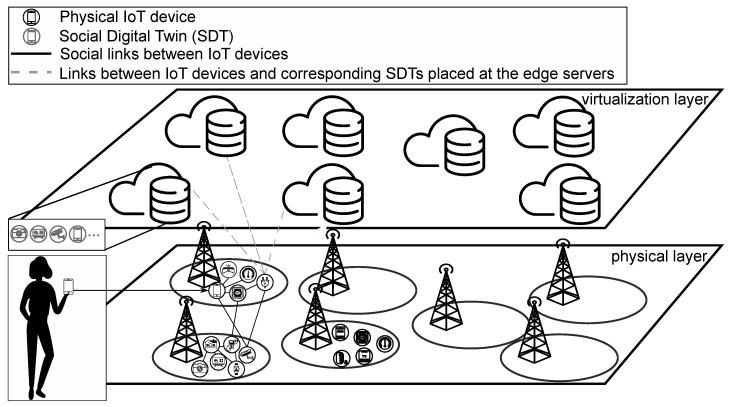
Layered Internet of Things (IoT) architecture.

In alignment with the existing literature [35,36], we consider the reference layered IoT architecture shown in Figure 1. The bottom layer accommodates physical IoT objects belonging to the real world (e.g., wearable devices, smartphones, sensors, actuators, etc.). These IoT objects are connected with each other and with other entities through the connectivity facilities offered by the 5G infrastructure.

The upper layer is the *virtualization layer*, where the digital representations of physical devices, i.e., the SDTs, are deployed. The SDT augments the physical device with storage and computing capabilities like DTs typically do. More in detail, it provides caching and preliminary filtering/aggregation of raw data streamed by the corresponding IoT device, before feeding IoT applications processing them. In addition to the semantic description of the corresponding physical device, the SDT also keeps the information about all the social relationships established by the corresponding physical device according to the SIoT paradigm [3]. In particular, the SDT stores metadata describing the type of friend devices and the SIoT relationship type for each friend device.

An IoT device willing to query friend devices, to discover services and/or push data to them, needs to read the friendship information stored in the SDT. Once such a piece of information is retrieved, the SDT itself can interact with its peers on behalf of the physical device. Then, SDTs of all (a subset of) friend devices can be contacted one-by-one, according to what we refer to as *friends browsing*.

We emphasize that SDTs are instantiated only for the most powerful devices (e.g., smartphones, tablets) able to connect to the Internet directly, which act as *head* devices. More constrained devices (e.g., wearable sensors, health, and fitness trackers), which are constrained in memory, computational capacity, and power, instead, may be associated with the SDT of the corresponding *head* device.

We assume that SDTs are deployed as virtualized applications, e.g., as containers [59] and instantiated in edge servers.

### 3.2. System Model

As illustrated in Figure 1, we consider an edge infrastructure consisting of *M* edge servers associated with wireless access points (e.g., a BS, a Wi-Fi AP) covering a specific area where *N* static IoT devices are located and establish relationships according to the SIoT paradigm. One SDT is associated to each IoT device. Since SDTs can store data and perform some processing, they have both CPU and storage demands that have to be taken into account when deploying them in an edge server, which has typically a finite amount of resources. Explicitly, we define the parameter Γ, Γ≥1, which limits the number of SDTs that can be deployed in an edge server; this number is the same for all edge servers.

The social network of IoT devices is represented by the graph $G_{P}=\left(V_{P},E_{P}\right)$. The set of vertices $V_{P}$ of graph $G_{P}$ corresponds to the physical devices connected by links in the set $E_{P}$; the links represent social relationships between devices. The weight of a link between physical devices *i* and *j* is denoted as $w_{ij}$. It tracks the information about the social relationship(s) between two physical devices. In particular, $w_{ij}$ is a binary variable and is given by:

(1) $$w_{ij}=\left\{\begin{array}{cc} 1, & \mathrm{if}\;\mathrm{device}\;i\;\mathrm{has}\;a\;\mathrm{social}\;\mathrm{relationship}\;\mathrm{with}\;\mathrm{device}\;j\;\mathrm{according}\;\mathrm{to}\;\mathrm{SIoT}\\ 0, & \mathrm{otherwise}. \end{array}\right.$$

The edge network is represented by the graph $G_{S}=\left(V_{S},E_{S}\right)$, where $V_{S}$ is a finite set of edge servers intended to host SDTs, whereas $E_{S}$ is a set of links between the edge servers. We reasonably assume that the number of IoT devices is larger than the number of edge servers, $|V_{P}\left|=N>\right|V_{S}|=M$, which does not limit the generality of the presentation.

The latency between each pair of edge servers, which represents the weight of the link between two vertexes *k* and *l* ($k,l\in V_{S}$) is given by $L_{kl}$. Similarly, we denote the latency between node $i\in V_{P}$ and node $k\in V_{S}$ as $L_{ik}$, and it corresponds to the latency between the device and its SDT. The latency depends on the distance between the involved nodes; more details will be given in Section 5.

## 4. Optimization Problem

### 4.1. Problem Definition

**Figure 2:**
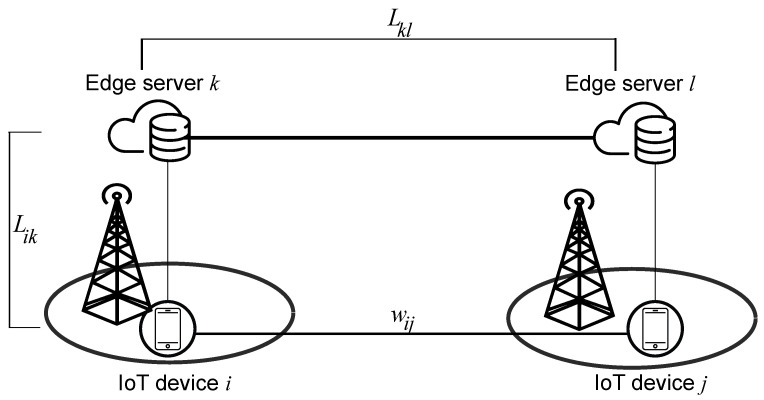
The cost function to be minimized includes the latency between the device and its SDT placed at the edge ($L_{ik}$) and the latency between a couple of friend SDTs placed at edge servers ($L_{kl}$).

We aim to allocate SDTs at edge servers so that a cost function is minimized which is expressed as a combination of (i) latency of connections between physical devices and their virtual representatives and (ii) latency between SDTs of physical devices linked by social relationships, while meeting a set of constraints on communication latency and capabilities of edge servers (see Figure 2, where the SDTs of two friend IoT devices, *i* and *j*, are deployed at edge server *k* and *l*, respectively).

We first formulate the SDT placement at the network edge as an MINLP optimization problem. We then transform the objective function by linear relaxation and proceed with the optimization problem using MILP.

To model the problem of SDTs placement on the given set of edge servers, we introduce a decision variable, $x_{ik}\in \left\{0,1\right\}$, which indicates whether the SDT of device *i* is assigned to edge server *k*. The binary variable $x_{ik}$ is equal to 1, if the SDT of device *i* is placed at edge server *k*, otherwise $x_{ik}=0$.

The mathematical problem can be formulated in terms of integer programming as follows:

(2) $$\min_{x}\sum_{i\in V_{P}}\sum_{k\in V_{S}}x_{ik}L_{ik}+\sum_{i\in V_{P}}\sum_{k\in V_{S}}\sum_{j\in V_{P}}\sum_{l\in V_{S}}x_{ik}x_{jl}w_{ij}L_{kl},$$

subject to

(3) $$\begin{array}{c} \sum_{k\in V_{S}}x_{ik}=1,\;\forall \;i\in V_{P}, \end{array}$$

(4) $$\begin{array}{c} \sum_{i\in V_{P}}x_{ik}\leq \Gamma ,\;\forall \;k\in V_{S}, \end{array}$$

(5) $$\begin{array}{c} L_{ik}\leq L_{\max},\;\forall \;i\in V_{P},\;\forall \;k\in V_{S}, \end{array}$$

(6) $$\begin{array}{c} x_{ik},x_{jl}\in \left\{0,1\right\},\;\forall \;i,j\in V_{P},\;\forall \;k,l\in V_{S}. \end{array}$$

Constraint (3) holds the condition that the SDT of device $i\in V_{P}$ can be assigned to one edge server only. Constraint (4) means that the maximum number of SDTs that can be deployed at an edge server $k\in V_{S}$ is limited by Γ. Constraint (5) includes a limitation on the latency between the physical device and the edge server hosting the corresponding SDT, which is upper bounded by $L_{\max}$. Finally, Constraint (6) reminds that we conveniently model the placement problem through binary variables.

### 4.2. Complexity Analysis

**Theorem** **1.** *Social digital twins placement problem is NP-hard.*

**Proof.**  We prove the SDT placement problem’s hardness by reducing it from the quadratic assignment problem (QAP), well known to be NP-hard [60].

*Quadratic Assignment Problem:* QAP solves the placement problem of a set of facilities to a set of locations with (i) the cost being a function of the distance and flow between the facilities and (ii) the cost associated with the placement a facility at a location. The optimization objective is to assign each facility to a location minimizing the total cost. Providing that *n* is the number of facilities and locations, N={1,2,...,n}, the facilities’ placement is given by the bijection N→N, i.e., a facility can be assigned to one location, and a location can accommodate one facility only.

*Social-Aware Closest Edge Placement problem:* SoCEP represents the relaxed version of QAP, wherein the requirement of objectivity and surjectivity of mapping the set of facilities (SDTs) to the set of placement positions (edge servers) is removed. We consider the problem of allocating SDTs with (i) the cost being a function of the latency $L_{kl}$ between edge servers and weight of social connections $w_{ij}$ between the IoT devices (see Figure 2) and (ii) the placement cost associated with SDTs of IoT devices being placed at edge servers. In our model, *N* SDTs are assigned to *M* edge servers such that SDTs corresponding to friend IoT devices are placed closer with the maximum possible proximity between SDTs and their IoT devices while allowing some flexibility for selecting/not selecting edge servers. More specifically, in comparison with QAP, $w_{ij}$ can be correlated with the flow between a couple of facilities, $L_{kl}$ can be associated with the distance between a couple of locations, and $L_{ik}$ can be closely related to the cost of placing facilities at locations.

This is a formulation of the quadratic assignment problem, which is NP-hard. ☐

### 4.3. Linearization

As one may deduce that there is a nonlinearity in the cost function in Equation (2) due to the quadratic form. To remove the nonlinearity, we perform the linearization of the objective function by introducing a new binary variable, $y_{ikjl}$, that equals 1 if SDTs of devices *i* and *j* are placed at edge servers *k* and *l*, respectively [61,62], i.e.,:

(7) $$y_{ikjl}=\left\{\begin{array}{cc} 1, & \mathrm{if}\;x_{ik}=1\;\mathrm{and}\;x_{jl}=1,\\ 0, & \mathrm{otherwise}. \end{array}\right.$$

We also denote the cost contributions related to the latency between the SDTs of IoT devices *i* and *j* placed at edge server *k* and *l*, respectively, as $C_{ikjl}$, by replacing $w_{ij}L_{kl}$.

Then, it is straightforward to verify that the cost function may be rewritten as:

(8) $$\min_{x,y}\sum_{i\in V_{P}}\sum_{k\in V_{S}}x_{ik}L_{ik}+\sum_{i\in V_{P}}\sum_{k\in V_{S}}\sum_{j\in V_{P}}\sum_{l\in V_{S}}y_{ikjl}C_{ikjl},$$

by satisfying the following constraints:

(9) $$\begin{array}{c} \sum_{k\in V_{S}}x_{ik}=1,\;\forall \;i\in V_{P}, \end{array}$$

(10) $$\begin{array}{c} \sum_{i\in V_{P}}x_{ik}\leq \Gamma ,\;\forall \;k\in V_{S}, \end{array}$$

(11) $$\begin{array}{c} \sum_{l\in V_{S}}y_{ikjl}=x_{ik},\;\forall \;k\in V_{S},\;\forall \;i,j\in V_{P}, \end{array}$$

(12) $$\begin{array}{c} \sum_{k\in V_{S}}y_{ikjl}=x_{jl},\;\forall \;l\in V_{S},\;\forall \;i,j\in V_{P}, \end{array}$$

(13) $$\begin{array}{c} L_{ik}\leq L_{\max},\;\forall \;i\in V_{P},\;\forall \;k\in V_{S}, \end{array}$$

(14) $$\begin{array}{c} x_{ik},x_{jl},y_{ikjl}\in \left\{0,1\right\},\;\forall \;i,j\in V_{P},\;\forall \;k,l\in V_{S}. \end{array}$$

Constraints (11) and (12) make it possible to take into account mutual social connections between a couple of SDTs. It indicates that SDTs of friend devices *i* and *j* can be allocated at the corresponding edge servers *k* and *l* only if the corresponding binary variables $x_{ik}$ and $x_{jl}$ are equal to 1. Constraint (13) bounds the latency between the physical device and the edge server hosting the corresponding SDT. Constraint (14) ensures that $x_{ik},x_{jl},y_{ikjl}$ are binary variables.

## 5. Performance Evaluation

In this section, we numerically elaborate on the performance of the proposed framework. First, we describe the simulation environment, which accepts the input parameters listed in Table 2, and characterize the baseline solution of interest by also describing the considered metrics. Then, we evaluate our model via simulations by using the IBM ILOG CPLEX Optimization Studio 12.10.0 software suite (to use branch-and-bound algorithm to solve the integer linear programming model) [63,64] on an Intel(R) Xeon(R) CPU E5-2620 v4 at 2.10 GHz with 19.7 GB RAM.

### 5.1. Simulation Scenario

**Figure 3:**
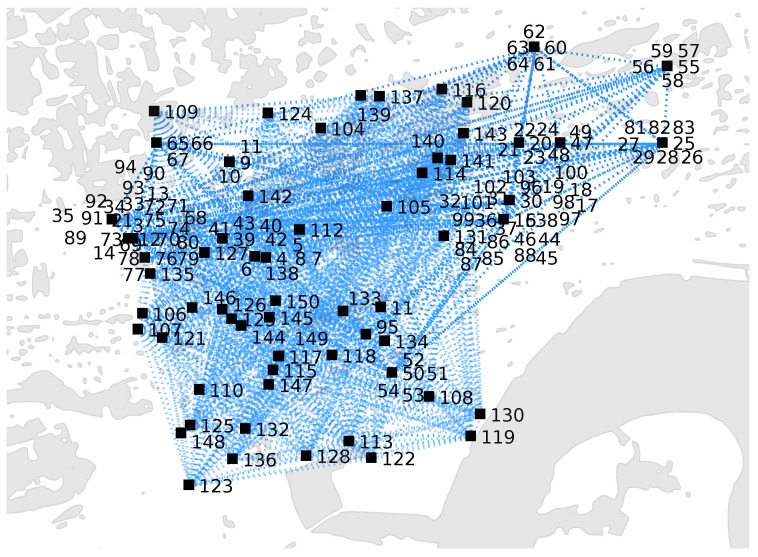
Snapshot of the considered dataset: black squares denote the IoT devices in the area, whereas the links indicate social interconnection between a couple of devices.

The simulation scenario, as shown in Figure 3, corresponds to the city center of Santander (Spain). The settings we used, in terms of area of interest and object’s metadata, are detailed in [66]. We restrict our analysis to an area that is roughly 4 km × 4 km (including uninhabited zones). Here, we assume that M=9 base stations are deployed. Each BS is located at the center of the area and has a coverage of 1 km^2^. An edge server is associated with each BS. We assume there are N=150 static IoT devices inside the area (which corresponds to public devices from the SIoT dataset (http://www.social-iot.org)).

From the dataset described in [66], we extract real IoT objects’ information (IDs, types, positions, and adjacency matrix with the social relationships). Here, value $w_{ij}$ defines the existence of a social relationship between a couple of devices, Equation (1). In this simulation, the set of relationships is given by R∈{POR} [3], where POR represents *Parental Object Relationship* (relationship among objects belonging to the same production batch) [3]. The average number of social relationships per IoT device is 80.

Similarly to [23,24], the latency $L_{ik}$ between device *i* and edge server *k*, as well as the latency $L_{kl}$ between edge serves *k* and *l* are estimated to be proportional to the distance between them. More precisely, $L_{ik}=\epsilon d_{ik}$ and $L_{kl}=\epsilon d_{kl}$, where ϵ is the distance to latency mapping coefficient, $d_{ik}$ and $d_{kl}$ are physical distances between device *i* and edge server *k* and between edge servers k,l, respectively.

The following assumptions also hold in our study: (i) when the edge server *k* that hosts the SDT is colocated to the same BS which its corresponding device, *i*, is connected to, then $d_{ik}=0$, $L_{ik}=0$; and (ii) when two SDTs are deployed at the same edge server (i.e., $d_{kl}=0,\forall k=l$), then $L_{kl}=0$. The first assumption is common in the literature [32] and allows to neglect the latency experienced over the radio interface. The second one neglects the intraedge server delay.

### 5.2. Benchmark

We compare the proposed model with a baseline solution, we refer to it as Closest Edge Placement (CEP), according to which SDTs of IoT devices are placed at the nearest edge server, neglecting social features. Hence, the CEP optimization problem can be expressed by:

(15) $$\min_{x}\sum_{i\in V_{P}}\sum_{k\in V_{S}}x_{ik}L_{ik},$$

with the same constraints as the SoCEP optimization problem.

### 5.3. Metrics

The following metrics are defined for the sake of evaluation:

- *Average latency among SDTs of friend IoT devices.* We measure this metric as the average latency between each couple of edge servers hosting SDTs whose corresponding physical devices are friends.
- *Average latency for friends browsing.* This metric includes both the latency between an IoT device and the edge server hosting the corresponding SDT plus the latency for browsing, one-by-one, all friend devices’ SDTs hosted in the edge infrastructure. The latter contribution is computed as the sum of the latencies experienced by an SDT to reach the edge servers hosting the SDTs of all friend devices, as read in the SDT table. The metric is used as an evaluation criterion for the proposed optimization model as it preserves all the kinds of latency contributions considered in Equation (8). It also allows one to figure out how the SDT placement affects the time needed for an IoT device to reach all friend devices, via the corresponding SDTs.

The second metric is especially relevant to assess the effectiveness of the service discovery procedure that implies considering friendship chains. Browsing the social network implies to “hop” from one SDT to the one of a friend of its, and so on. For instance, some applications may need to push (query) data to (from) categories of friends. It is the case of a given software patch that needs to be safely delivered to all the devices or sensors of the same brand/batch.

Reported results for both metrics refer to the solution of a specific instance of the optimization problem (both CEP and SoCEP) with inputs and settings as described before.

### 5.4. Simulation Results

In this section, we examine the performance of the proposed placement solution, SoCEP, against the benchmark scheme, CEP, when varying the maximum latency, $L_{\max}$, in the range 0–14 ms and the edge server’s capacity, Γ, in the range 20–60.

**Figure 4:**
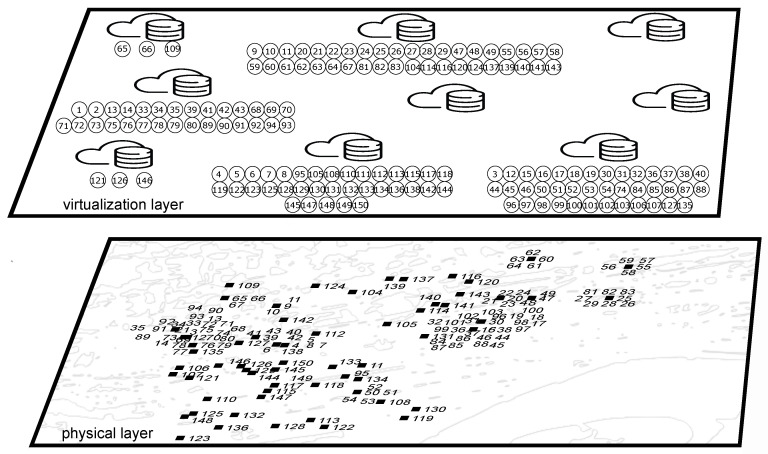
SDTs placement in the edge infrastructure for SoCEP with $\Gamma =40,L_{\max}=5$ ms.

Figure 4 reports one instance for the SoCEP solution, corresponding to the following settings: edge server’s capacity, Γ, equal to 40, and maximum latency between IoT devices and edge servers, $L_{\max}$, equal to 5 ms. At the bottom of the Figure, physical IoT devices are shown in the map, whereas at the top of the Figure, the placement of the corresponding SDT among the nine available edge servers is reported.

**Figure 5:**
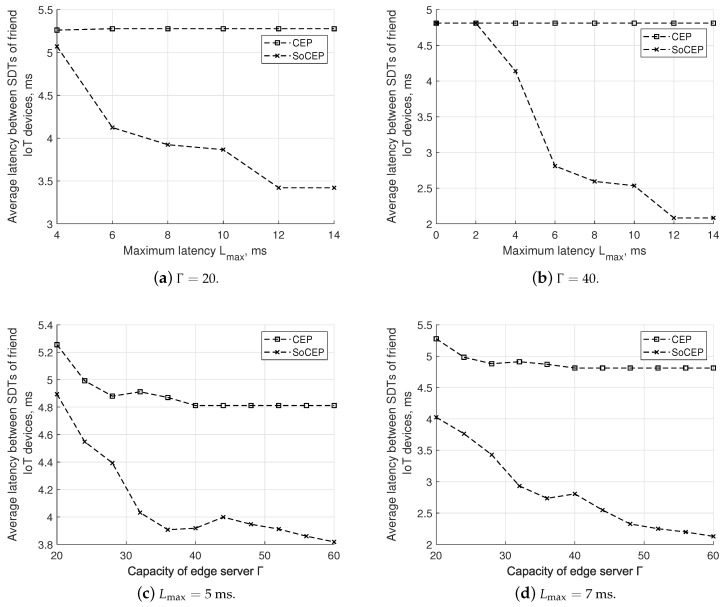
SoCEP vs. CEP: average latency between SDTs of friend IoT devices.

In Figure 5, we report the average latency between SDTs of friend IoT devices. It can be observed that the proposed solution performs significantly better than the benchmark solution with gains up to 35.1 and 56.7% for $L_{\max}$ = 14 ms (as illustrated in Figure 5a,b, respectively) and up to 20.6 and 55.7% for Γ=60 (as demonstrated in Figure 5c,d).

The metric decreases for SoCEP as the latency constraint is relaxed. In this case in fact, relaxing the constraint of the proximity of SDTs with their physical counterpart allows SDTs of friend IoT devices to be placed closer. The poorer performance (i.e., higher latency values) of the CEP scheme has to be ascribed to the fact that it attempts to place SDTs closer to their physical counterparts, while being oblivious of social relationships among IoT devices.

It should be further noticed that for Γ = 20 (Figure 5a) no optimal solution is found by solving (8) and (15) for $L_{\max}<4$ ms, since edge servers cannot accommodate the SDTs for all the IoT devices covered by the colocated BS.

As the capacity of the edge servers increases, SoCEP provides lower latency (as shown in Figure 5c,d) as a consequence of the fact that SDTs of friend devices are likely placed either in the same edge server or in one server in close vicinity (Figure 5b). In these cases, one may observe some fluctuations of SoCEP curves. The tightness of social communities among IoT devices (or among SDTs) can explain this behavior. Indeed, even slight relaxation of the capacity constraint may notably change the SDT placement scheme.

**Figure 6:**
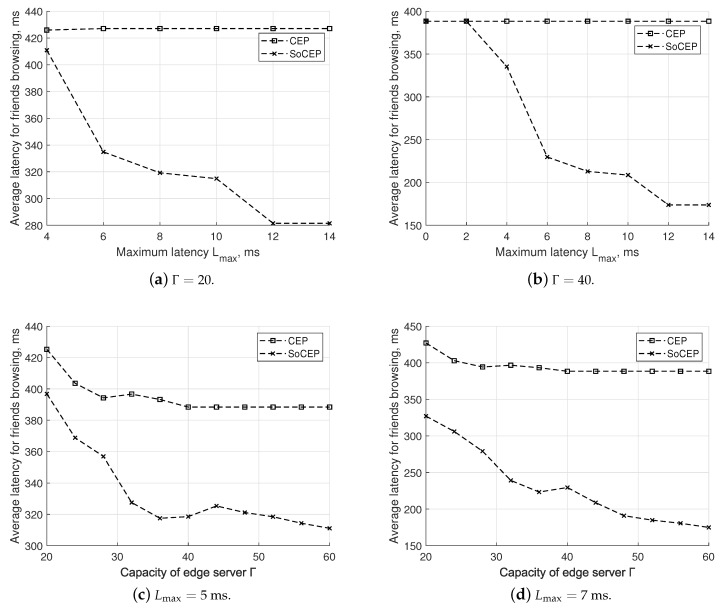
SoCEP vs. CEP: average latency for friends’ browsing.

Finally, in Figure 6, we examine the effectiveness of the proposal in providing low latency in browsing the friends by the SDTs. Results confirm that the proposal is quicker in the browsing procedure compared to CEP. Delay values are more than halved when the maximum latency $L_{\max}$ exceeds 10 ms and Γ=40. This finding is quite crucial as it proves that accounting for social features in the SDT placement allows one to significantly improve the browsing procedure.

### 5.5. Computation Time

We solve the problem optimally by applying a branch-and-bound algorithm, which is a variation of the exhaustive search method by considering an acceptable solution set. The computational complexity of such precise algorithms is generally exponential. The problem complexity grows with the increase in the problem size. Moreover, we emphasize that not only the problem size but also its constraints affect the computational complexity. For instance, in Table 3, one may observe that the relaxation on the maximum latency, $L_{\max}$, slows down the process of finding optimal solutions. Such a trend can be explained by the increase in the number of acceptable options for placing SDTs.

Clearly, the reported values for the computation time become unacceptable for less stringent latency constraints. Hence, as a future work we target the design of an efficient (in terms of computation time) heuristic algorithm that can solve the problem in large networks by targeting the SoCEP objectives with a little compromise in the solution optimality.

In our proposal, we assume that the optimal algorithm, and then also the heuristics, for the placement of SDTs in edge servers is implemented in a centralized manner by the orchestrator of the edge infrastructure. In the case of an edge infrastructure compliant with the European Telecommunications Standards Institute (ETSI) MEC specifications, the placement of SDTs would be decided by the *ME orchestrator* [67] handling a certain number of edge servers.

## 6. Conclusions and Future Works

In this paper, we have presented a cutting-edge solution for digital twins placement at the edge, which accounts not only for proximity requirements but also for relationships among IoT devices established according to the SIoT paradigm. We have formulated the proposal as an optimization problem and obtained promising results. Indeed, the proposal can actually contribute to make the SIoT more viable, by reducing the time for the browsing procedures.

The proposal has some room for improvements. First, the type and the number of social relationships can be considered in the social graph and to derive the cost function by differently weighing the latency contributions between SDTs of friend devices. Indeed, some types of friend devices may require the corresponding SDTs to interact more quickly than others.

Second, more intelligent placement strategies that take into account the specifics of the network deployment, the types of IoT devices, and the mobility patterns are of special interest for future research contributions. For instance, the mobility patterns can be considered in the formulation of the optimization algorithm as proposed in [24] and, in addition, the placement decision can be dynamically modified when the latency constraints do not comply anymore with the required ones for the connectivity between physical IoT devices and their digital counterparts. In this case, the challenge of running in real-time the replacement of digital twins is not only related to the need of rerunning the optimization algorithm but also to the migration of the SDT from one edge server to another (see, e.g., [68]). How to achieve seamless migration among edge servers is still an open issue in the literature which deserves to be investigated.

## Figures and Tables

**Table 1:** Description of notations.

Parameter	Description
$G_{P}\left(V_{P},E_{P}\right)$	Weighted undirected graph of physical IoT devices
$v_{P}\in V_{P}$	Set of physical IoT devices
$e_{P}\in E_{P}$	Set of links between physical IoT devices
$G_{S}\left(V_{S},E_{S}\right)$	Weighted undirected graph of edge servers
$v_{S}\in V_{S}$	Set of edge servers
$e_{S}\in E_{S}$	Set of links between edge servers
$L_{ik}$	Latency between physical device $i\in V_{P}$ and its SDTs placed at edge server $k\in V_{S}$
$L_{kl}$	Latency between edge servers $k,l\in V_{S}$
$C_{ikjl}$	Cost of connections between devices $i,j\in V_{P}$ and their SDTs placed at edge servers $k,l\in V_{S}$
Γ	Capacity of an edge server (maximum number of SDTs to be stored per edge server)
$L_{\max}$	Maximum latency between a physical device and its SDT
$d_{ik}$	Physical distance between IoT device *i* and edge server *k* (that hosts its SDT)
$d_{kl}$	Physical distance between SDTs deployed at edge servers k,l
**Binary Variable**	**Description**
$w_{ij}=1$	Physical IoT device $i\in E_{P}$ is connected to device $j\in E_{P}$
$x_{ik}=1$	SDT of device $i\in V_{P}$ is mapped to edge server $k\in V_{S}$
$y_{ikjl}=1$	SDT of device $i\in V_{P}$ is mapped to edge server $k\in V_{S}$ and SDT of device $j\in V_{P}$ is assigned to edge server $l\in V_{S}$

**Table 2:** Main simulation parameters.

Parameter	Value
Number of IoT devices, *N*	150
Number of edge servers, *M*	9
Capacity of an edge server, Γ	var
Maximum latency between a devices and an edge server, $L_{\max}$	var
Distance to latency mapping coefficient, ϵ	3.33 ms/km [65]

**Table 3:** Computation time.

$L_{\max}$	3 ms	3.5 ms	4 ms	4.5 ms	5 ms	5.5 ms	6 ms	6.5 ms	>7 ms
**Time**	1.6 s	3.7 s	5.8 s	7.1 s	46.8 s	1534.4 s	2601.5 s	2718.4 s	4380.9 s
